# Index of Orthodontic Treatment Need in Obese Adolescents

**DOI:** 10.1155/2015/876931

**Published:** 2015-04-05

**Authors:** Maria Rita Giuca, Marco Pasini, Silvia Caruso, Simona Tecco, Stefano Necozione, Roberto Gatto

**Affiliations:** ^1^Department of Surgical, Medical, Molecular and Critical Area Pathology, Unit of Pediatric Dentistry, University of Pisa, Via Roma 67, 56126 Pisa, Italy; ^2^Department of Life, Health and Environmental Sciences, University of L'Aquila, Coppito Piazzale Salvatore Tommasi 1, 67100 L'Aquila, Italy; ^3^University Vita-Salute San Raffaele, Dental Clinic, Via Olgettina 60, 20132 Milano, Italy

## Abstract

*Aim*. This case-control retrospective study is aimed at assessing if obese adolescents need more orthodontic treatment in comparison with normal-weight patients of the same age. *Methods*. The test group included 100 obese subjects (50 males and 50 females; average age: 13.09 ± 1.19 years old) and the control group included 100 normal-weight patients matched for age and sex (50 males and 50 females; average age: 13.07 ± 1.26 years old). Clinical examinations were conducted on dental casts to assess the need of orthodontic treatment, by using the Index of Orthodontic Treatment Need (IOTN) (DHC, dental health component; AC, aesthetic components). *Results*. No statistically significant difference (*P* > 0.05) was observed between the two groups with regard to AC. Obese females showed a significant (*P* < 0.05) higher percentage of DHC 3 (32%) in comparison to the normal-weight girls (22%); for the other grades of DHC and for the single kind of malocclusion, no significant difference was found. *Conclusions*. Obese adolescents showed a similar need for orthodontic treatment compared to normal-weight patients of the same age. However, in obese females, a slightly greater need for orthodontic treatment was observed, compared to normal-weight patients.

## 1. Background

Obesity is nowadays an increasingly growing health problem, even in childhood and adolescents: the prevalence of childhood obesity in Italy ranged from 7.5% in the North to 16.6% in the South [1].

It is an abnormal accumulation of body fat, usually 20% or more over an individual's ideal body weight and it is often accompanied by an increased risk of morbidity and mortality [2]. Obesity is often associated with poor eating habits or excessive supply of food and a sedentary lifestyle; however, obesity can also be linked to genetic, environmental factors, hormonal dysfunctions, and the psyche of the patient [3, 4].

There are two forms of obesity: primary and secondary. The primary is due to genetics, nutrition, and behavioural and psychosocial factors that lead to an imbalance between food intake and energy expenditure. Secondary obesity is linked to specific pathological conditions such as Cushing's syndrome, hypothyroidism, insulinoma, the Stein-Leventhal syndrome, some forms of diabetes mellitus, use of certain drugs, and hypothalamic endocrine disorders [5, 6].

Because obesity can be considered as a systemic disease that predisposes to a variety of comorbidities and complications that affect overall health, people who are obese require a multidisciplinary approach [7]. Moreover, it was suggested that childhood obesity may affect also the maxillofacial morphology of growing patients [8].

An advanced craniofacial growth, measured by the carpal analysis and cervical vertebral maturation method, was found in young obese patients in comparison to normal-weight patients; thus, the authors stated that an earlier examination and orthodontic treatment in obese children might be necessary compared to normal-weight patients [9].

Leptin, a hormone mainly produced by white adipose tissue for controlling appetite and the build-up of reserves in the form of adipose tissue, might be directly involved in the process of skeletal growth because it accelerates the production of gonadotropin-releasing hormone (GnRH) in the hypothalamus and it has an effect on adenohypophysis, accelerating pubertal development. Obese patients exhibited higher levels of leptin that may influence the onset of malocclusions [10].

Furthermore, it has been observed that obese patients showed a different craniofacial morphology in comparison to normal-weight children, with an increased maxillary and mandibular size, greater maxillary and mandibular prognathism, a less convex profile, and a smaller mandibular plane and lower anterior and posterior face height [11].

Thus, an earlier skeletal maturation and the presence of abnormal cephalometric values, compared with the standard values, could affect the need for orthodontic treatment for young obese patients in a growth phase. To determine the need for orthodontic treatment according to the degree of malocclusion, several indices were introduced [12, 13], for example, the Index of Orthodontic Treatment Need (IOTN). This index has been introduced by Brook and Shaw [14] and it is one of the most commonly used indexes, whose validity and reliability have been verified in literature [15].

The purpose of this study was to evaluate if obese adolescents aged between 11 and 15 years old have a higher degree of malocclusion and an increased need for orthodontic treatment compared with normal-weight patients of the same age. The null hypothesis is that there is no difference between these two samples in terms of orthodontic treatment need, evaluated by using the IOTN.

## 2. Materials and Methods

### 2.1. Study Population

In this retrospective study, the dental casts of 100 obese and 100 normal-weight patients, who had been reported to the university orthodontic clinic and who were selected from the 1500 available models, were randomly selected. The study was conducted from January to December 2014.

The study was based on an estimated sample size of 200 subjects, with a ratio 1 : 1 for the cases and controls, which has been calculated to be adequate to achieve 90% power with an alpha error of 0.05 to detect a difference of 25% of proportions of IOTN, ≤2 between cases and controls.

Inclusion criteria were as follows: age between 11 and 15 years old, permanent dentition, available dental casts, Caucasian and Italian nationality, and informed consent signed from parents or guardians.

The exclusion criteria were patients who had received previous orthodontic treatment, patients who had reported bad habits as sucking fingers, atypical swallowing, mouth breathing, pacifier use beyond 3 years, craniofacial anomalies or syndromes, medications that may have influenced the craniofacial growth, and non-Caucasian and non-Italian patients.

All procedures were conducted according to the principles expressed in the Declaration of Helsinki (1964).

An investigator approached parents or guardians and explained the aim of the study and its procedure; all parents and caregivers gave their consent.

The patients were randomly selected from the records of the patients referred to the university orthodontic clinic and a random computerized analysis was carried out in order to select the patients.

To allocate the patients in the test (obese) and control (normal weight) groups the BMI (body mass index) was calculated (weight in kilograms divided by height in meters squared) according to the International Obesity Task Force classification.

The BMI of adults remains relatively constant unless they gain or lose a lot of weight and it is possible to use the same cutoffs for the definition of obesity, underweight and overweight, regardless the age and sex. However, children BMI changes as they mature and these patterns of growth differ between males and females. Therefore, each body mass index value was matched to the corresponding percentile on the international charts according to the subject's age and sex to calculate the standard deviation score of the subject's BMI (BMI SDS: standard deviation score of patient's body mass index) [16].

The test group included 100 obese subjects (50 males and 50 females; average age: 13.09 ± 1.19 years old).

The control group included 100 normal-weight patients matched for age and sex (50 males and 50 females; average age: 13.07 ± 1.26 years old).

The BMI SDS was 2.6 ± 1.6 in the test group and 0.6 ± 0.3 in the control group.

### 2.2. Dental Cast Examination

Dental cast examinations were conducted to assess the need of orthodontic treatment, by using the Index of Orthodontic Treatment Need (IOTN) developed by Brook and Shaw [15, 17] and this index was the primary outcome of the study.

This index has both an aesthetic component (AC) and a dental health component (DHC).

The aesthetic component (AC) consists of a scale of 10 colored pictures showing different levels of beauty of the smiles and the objective of the scale is to find a similar occlusion or the level of equivalent severity to the individual appraised, placing the occlusion in relation to the number where “1” represents the most attractive occlusion and “10” represents the least attractive occlusion. An AC score below “5” indicates no need or only a slight need of orthodontic treatment; a score between 5 and 7 shows a borderline need; and a score between 8 and 10 shows a definite need [18]. Orthodontists matched the patients to these photographs. The DHC registers the occlusal characteristics of a malocclusion and only the highest scoring trait is used to assess treatment need: increased overjet, increased overbite, open bite, unilateral posterior cross bite, anterior cross bite, bilateral posterior cross bite, upper crowding, lower crowding, and impeded eruption [19].

There are five different levels, from degree 1 (no need for orthodontic treatment) up to degree 5 (great need for orthodontic treatment) (Table 1).

The same blind investigator, who had received extensive training and was calibrated to use the IOTN and BMI SDS, performed all clinical examinations; the investigator was unaware of group assignment.

Each study model was trimmed with a model trimmer to articulate the maxillary and mandibular arches in occlusion. The examination lasted approximately 20 minutes per dental cast; no radiographs or previous written records were used.

## 3. Statistical Analysis

Each model was evaluated twice, with an interval of 2 weeks between the measurements, by the same operator.

Intraobserver variability was calculated by using Cohen's Kappa coefficient. The coefficients obtained ranged between 0.93 and 0.98. We assessed statistically significant differences between the two groups in the prevalence (percentage) of each IOTN score in the two groups by using Chi-square test and Fisher test. Odds ratio (OR) was evaluated and the 95% confidence interval (CI) was used to estimate the precision of the OR.

Wilcoxon test was used to evaluate the total of AC and DHC values between the two groups.

The level of significance was set at *P* < 0.05.

The statistical analysis was performed with the SPSS (Statistical Package for Social Sciences, Chicago, USA) 22.0 program.

## 4. Results

Table 2 shows the results of the AC Index.

No statistically significant difference (*P* > 0.05) was observed between the test group and the control group with regard to the aesthetic component of the index. The obese subjects and the normal-weight adolescents exhibited similar AC degrees 1–4 (33% in the test and 36% in the controls), 5–7 (44% in the test and 41% in the controls), and 8–10 (23% both in test and controls) (*P* > 0.05).

No significant difference was found between the two groups according to the sex difference for the AC (*P* > 0.05). The evaluation of the total of AC ranks (average score of 101.65 in tests and 99.34 in controls) did not show a significant difference between the two groups (*P*: 0.38, *P* > 0.05).

Regarding the dental health component of the index (DHC), it was found that patients of the test group showed a percentage of patients with a degree 1 (16%) similar (*P* > 0.05) to that in the control group (19%); furthermore, with regard to the prevalence in males and females, there were no significant differences (*P* > 0.05) between the two groups (Table 3).

Obese patients showed a lower frequency of degree 2 compared to the control group; however, the difference between the two groups was not statistically significant (*P* > 0.05). It was then observed, by separately analysing patients regarding degree 2, that females in the test group showed a lower percentage compared to normal-weight patients, but once again, difference was not significant (*P* > 0.05).

The test group showed a higher percentage of degree 3 compared to control group but statistical analysis did not find significant differences (*P* > 0.05). Also regarding this degree, the females of the obese group exhibited a higher degree 3 in comparison to the females of the control group and the difference was significant (*P* < 0.05).

On the contrary, males in the two groups showed the same percentages of DHC 3 (*P* > 0.05).

In the test group a slightly higher percentage of DHC 4 was found in the test group in comparison to the controls; however, the statistical difference was not significant (*P* > 0.05). It was then observed that, by separately analysing patients regarding degree 4, no significant differences were found (*P* > 0.05).

The percentage of DHC degree 5 was similar in the two groups and no significant difference (*P* > 0.05) was found between the test and the control groups.

The average score obtained by Wilcoxon analysis was 80 in tests and 95.19 in controls; the evaluation of the total of DHC ranks did not show a statistically significant difference between the two groups (*P*: 0.09, *P* > 0.05).

Regarding the type of malocclusion, the group of obese patients showed a lower prevalence of dental alveolar crowding in both maxillary and mandibular arches; however, the difference between the test group and control group was not significant (*P* > 0.05). Furthermore, for all other analysed malocclusions (increased overjet, increased overbite, open bite, unilateral posterior cross bite, anterior cross bite, bilateral posterior cross bite, and impeded eruption), obese subjects exhibited a higher percentage in comparison to the controls even if no significant difference in prevalence (*P* > 0.05) was observed between the two groups (Table 4).

## 5. Discussion

The IOTN is used to assess objectively the degree of malocclusion and to evaluate the aesthetic impact of malocclusion.

Furthermore, it is a useful tool for diagnostic and treatment orthodontic plan both in adults and in children [20]. We included patients aged between 11 and 15 years old because it is much easier and more accurate to use the IOTN in the late mixed/early permanent dentition stage rather than in the deciduous/early mixed dentition and because the multibrackets orthodontic treatment is carried out in the majority of cases in the late mixed/early permanent dentition stage. This study observed that obese adolescents show percentages similar to normal-weight ones regarding the need of orthodontic treatment, by analysing both AC and DHC. It would seem that obesity is not correlated with the severity of the malocclusion and orthodontic treatment need. However, obese females needed more orthodontic treatment regarding DHC 3, which may be associated with earlier pubertal development of females than males [21].

This result suggests that early orthodontic intervention, which aims to modify orthopaedic growth, for example, by using functional appliances, is often needed before the pubertal peak and that early therapy is intended as a preventive measure to avoid more complex treatment in the future and worsening case prognoses. In literature, it was observed that obese children exhibited a higher discrepancy between skeletal and chronologic ages according to the carpal analysis and for a significantly higher cervical vertebral maturation score, compared to normal-weight patients [9]. For these reasons, especially obese girls should be assessed early by an orthodontist in order to, if necessary, take advantage on the pubertal peak.

In fact, it was demonstrated that the orthodontic treatment carried out during or shortly after the pubertal peak allows for better and faster therapeutic results, as in the case of mandibular advancement for the correction of Class II skeletal [22].

Regarding the type of malocclusion, the group of obese patients showed a slightly lower prevalence of dental alveolar crowding in both upper and lower arches even if the difference was not significant. This observation is probably because obese children show a greater size of the skeletal base, as evidenced in literature (the anterior cranial base length and the maxillary length) allowing having more available space for the alignment of the teeth in both the upper arch and the lower arch [10, 23].

Must et al. observed that obese children show a precocious teeth eruption and this may influence dental sequencing and it could increase the risk of malocclusion, in particular in females that often show more erupted permanent teeth (canines, premolars, and second premolars) than males. On average, it was observed that obese children have 1 : 44 more erupted teeth than normal-weight patients, after adjusting for age, gender, and race ethnicity, because the study included patients of different ethnic groups; moreover, the socioeconomic status was not a confounder of the associations [24].

The increased risk of malocclusion can also lead to later temporomandibular disorders, periodontal problems, and hygiene problems, as the teeth with altered occlusion are more difficult to brush properly; however, the quick teeth eruption does not always lead to malocclusion, as it is determined by several genetic and environmental factors, in particular the bad habits, the use of the pacifier beyond the limits, mouth breathing, and atypical swallowing that could affect the development of the dental arches and also lead to severe alterations [10]. For this reason, in order to avoid possible confounders, the patients showing these risk factors were excluded from this study.

As for all other types of malocclusion, obese patients showed a higher percentage, although not significant, compared with normal-weight patients; some types of malocclusion such as the lateral and the anterior cross bite early require interceptive orthodontic therapies, about the eruption of the first permanent molars, to avoid the onset of permanent skeletal deformities, in particular in the case of the unilateral cross bite that can cause a skeletal lateral deviation; so it is important not to wait until the age of adolescence to begin orthodontic treatment. The compliance factor plays an important role because the removable appliances must be brought constantly and this is more easily achieved in children compared to the adolescence when the patient is usually less cooperative in carrying appliances.

As regards the comparison of these results with those of the paedodontic Italian population, a recent study [13] focused on a sample of Italian children, with an average age of 9.3 years; a high need of orthodontic treatment (86% of young patients showed a moderate or high risk of malocclusion, grades 3 and 4, resp.) was observed. In our study, in both the control and test groups, a lower percentage of patients with moderate to severe malocclusion (corresponding to grades 3 and 4 of DHC) was observed. However, the index used in our study was not the same one used in that study (ROMA index). Moreover, the inclusion criteria of our study excluded all patients who had been treated with any fixed or removable orthodontic appliances and included patients with a higher average age.

A limit of the study is that DHC degrees 1, 4, and 5 of the index have few subjects, and maybe that is why a significant difference could not be detected. On this point, it also should be emphasized that patients already treated with orthodontic treatments were excluded from the selection of the sample and patients in this study with severe orthodontic problems were supposed to be few.

Another limitation is that the study was conducted on dental casts and not directly on patients and this made it impossible to evaluate the sociopsychological aspects of the index, in particular in reference to the AC, which usually should be assessed directly by patients. In fact, obese patients may have a lower self-esteem of their body and of their teeth, while the data of our study did not show significant differences; in particular, the literature shows that the degree of malocclusion has a greater impact on the psychological level in girls [25]. However, this way, it was possible to avoid a subjective evaluation, and in some cases nonrealistic, by young patients who are not always able to assess appropriately their occlusion. Then, it should be highlighted that no similar study has ever been conducted in literature. The data resulting from this study cannot confirm that obesity in adolescent age certainly represents a risk factor for the onset or aggravation of a malocclusion.

## 6. Conclusions

The results demonstrate that obese adolescent patients and normal-weight patients show the same need for orthodontic treatment.

However, obese females exhibited a significant higher DHC 3 and a higher severity of malocclusion compared to the control subjects. No significant difference between the groups was observed regarding the AC and the type of malocclusion.

A correct knowledge of the characteristics of dentoskeletal malocclusions that can occur in young obese patients could represent a useful tool for the orthodontist in orthodontic diagnosis and treatment plan, in particular if functional orthopaedic therapies are needed before or during pubertal peak.

These results should be considered preliminary and need to be confirmed with further studies involving a larger population.

## Figures and Tables

**Table 1:** Description of the degrees of the IOTN (DHC score).

DHC score	Occlusion	Orthodontic treatment need
1	Perfect or almost perfect occlusion	No need
2	Occlusion with slight irregularities	Slight need
3	Occlusion with higher irregularities	Borderline need
4	Occlusion with more severe irregularities	High need
5	Severe dental health problems	Very high need

**Table 2:** Prevalence of the aesthetic component (AC) of the IOTN in the two groups, with the indication of significant differences.

Degrees of IOTN (AC)	Test group *N*: 100 patients	Control group *N*: 100 patients	Odds ratio Confidence interval 95%	*P* value
1–4				
Total	**33**	**36**	**0.88 (0.49–1.57)**	**0.65 N.S.**
Males	15	18	0.8 (0.38–1.7)	0.57 N.S.
Females	18	18	1 (0.49–2.1)	1 N.S.
5–7				
Total	**44**	**41**	**1.13 (0.64**–**1.98)**	**0.67 N.S.**
Males	23	21	1.12 (0.58–2.2)	0.73 N.S.
Females	21	20	1.06 (0.54–2.11)	0.86 N.S.
8–10				
Total	**23**	**23**	**1.0 (0.52**–**1.93)**	** 1 N.S.**
Males	12	11	1.1 (0.46–2.63)	0.82 N.S.
Females	11	12	0.91 (0.38–2.16)	0.82 N.S.

N.S.: not significant.

Significant differences in Chi-square test results with 95% confidence interval; *P* < 0.05.

**Table 3:** Prevalence of the IOTN (degrees 1–5) in the test and in the control group, with the indication of significant differences.

Degree of the IOTN	Test group *N*: 100 patients	Control group *N*: 100 patients	Odds ratio Confidence interval 95%	*P* value
Degree 1				
Total	**16**	**19**	**0.81 (0.39–1.69)**	**0.58 N.S.**
Males	8	10	0.78 (0.3–2.07)	0.62 N.S.
Females	8	9	0.88 (0.32–2.38)	0.8 N.S.
Degree 2				
Total	**21**	**32 **	**0.56 (0.3–1.07)**	**0.08 N.S.**
Males	13	15	0.85 (0.38–1.89)	0.68 N.S.
Females	8	17	0.42 (0.17–1–04)	0.05 N.S.
Degree 3				
Total	**32**	**22 **	**1.67 (0.89–3.14)**	**0.11 N.S.**
Males	14	14	1 (0.45–2.22)	1 N.S.
Females	18	8	2.52 (1.04–6.11)	0.03^∗^
Degree 4				
Total	**20**	**16**	**1.31 (0.63–2.71)**	**0.46 N.S.**
Males	10	7	1.48 (0.54–4.05)	0.45 N.S.
Females	10	9	1.12 (0.44–2.89)	0.81 N.S.
Degree 5				
Total	** 11**	**11 **	**1 (0.41–2.43)**	** 1 N.S.**
Males	5	4	1.26 (0.33–4.85)	0.73 N.S.
Females	6	7	0.85 (0.27–2.62)	0.77 N.S.

N.S.: not significant.

^∗^Significant differences in Chi-square test results with 95% confidence interval; *P* < 0.05.

**Table 4:** Prevalence of the types of malocclusion (DHC of the IOTN) in the test and in the control groups, with the indication of significant differences.

DHC	Test group *N*: 100 patients	Control group *N*: 100 patients	Odds ratio Confidence interval 95%	*P* value
Increased overjet	18%	14%	1.35 (0.63–2.89)	0.44 N.S.
Anterior cross bite	7%	4%	1.81 (0.51–6.38)	0.35 N.S.
Upper crowding	7%	14%	0.46 (0.18–1.20)	0.11 N.S.
Lower crowding	6%	13%	0.43 (0.16–1.17)	0.09 N.S.
Increased overbite	11%	7%	1.64 (0.61–4.42)	0.32 N.S.
Open bite	10%	7%	1.48 (0.54–4.05)	0.45 N.S.
Unilateral posterior cross bite	14%	11%	1.32 (0.57–3.06)	0. 52 N.S.
Bilateral posterior cross bite	8%	5%	1.65 (0.52–5.24)	0.39 N.S.
Impeded eruption	3%	2%	1.52 (0.25–9.27)	0.65 N.S.

N.S.: not significant.

Significant differences in Chi-square test results with 95% confidence interval; *P* < 0.05.
